# Effects of neuromuscular warm-up on athletes’ change-of-direction performance and knee isokinetic muscle strength: a systematic review and meta-analysis

**DOI:** 10.3389/fphys.2026.1750821

**Published:** 2026-03-19

**Authors:** Chenwen Zhu, Yunfei Lu, Meiling Tao, Tianyue Yin, Jiuzhang Li, Steve Thompson, Nan Gu

**Affiliations:** 1 China Table Tennis College, Shanghai University of Sport, Shanghai, China; 2 School of Athletic Performance, Shanghai University of Sport, Shanghai, China; 3 Discipline of Exercise and Sport Science, Faculty of Medicine and Health, The University of Sydney, Sydney, NSW, Australia; 4 School of Sport and Physical Activity, College of Healhaoth, Wellbeing and Life Sciences, Sheffield Hallam University, Sheffield, United Kingdom

**Keywords:** agility, athletic performance, elite athletes, injury prevention, training protocol

## Abstract

**Background:**

Neuromuscular warm-up is a structured protocol containing at least three of the following exercise types: resistance, dynamic stability, core strength, plyometrics, and agility. Neuromuscular warm-up holds significant clinical value for enhancing athletic performance and reducing injury risk. However, current evidence remains limited regarding its effects on change-of-direction (COD) performance and knee isokinetic muscle strength—two physical qualities critically associated with performance outcomes and injury prevention in multidirectional sports. Furthermore, a comprehensive synthesis is lacking on how to tailor warm-up protocols to optimally improve these two interrelated domains.

**Objective:**

This review aims to: 1) evaluate the effects of neuromuscular warm-up on COD performance and knee isokinetic muscle strength, and 2) systematically analyze moderating effects of warm-up protocols (number of exercise, frequency, sets, repetitions, duration, and metrics), athlete level, and study designs (randomized vs. non-randomized trials).

**Methods:**

Searches were conducted in PubMed, Web of Science (Core Collection), Embase, and Scopus on 5 May 2025, and updated on 15 May 2025. Pooled effects for each outcome were summarized using standardized mean difference (Hedges’ g) through a three-level meta-analysis model, subgroup and regression analyses were used to explore moderators. The certainty of evidence was assessed using the GRADE approach.

**Results:**

From 25,251 records, 19 studies (n = 810) were included, with a mean PEDro score of 6.00 (high quality). Neuromuscular warm-up significantly improved COD performance (g = 0.46 [0.09, 0.82], I^2^-2 = 33.7%; I^2^-3 = 37.7%, Moderate GRADE) and knee isokinetic muscle strength (g = 0.72 [0.39, 1.04], I^2^-2 = 69.5%; I^2^-3 = 5.2%, High GRADE) versus controls (regular or dynamic warm-up). Meta-regression analysis indicated that sets in neuromuscular warm-up protocols significantly moderate COD performance. Subgroup analyses indicated that warm-up protocols (neuromuscular warm-up frequency and metrics), athlete levels, and study designs significantly influenced both COD performance and knee isokinetic muscle strength (p < 0.05).

**Conclusion:**

Neuromuscular warm-up significantly enhances COD performance and knee isokinetic muscle strength compared to the control group, moderated by warm-up protocols (frequency, sets, and metrics), athlete level, and study designs.

**Systematic Review Registration:**

This study is registered with ClinicalTrials.gov as CRD420251046324.

## Graphical Abstract

**Figure:**
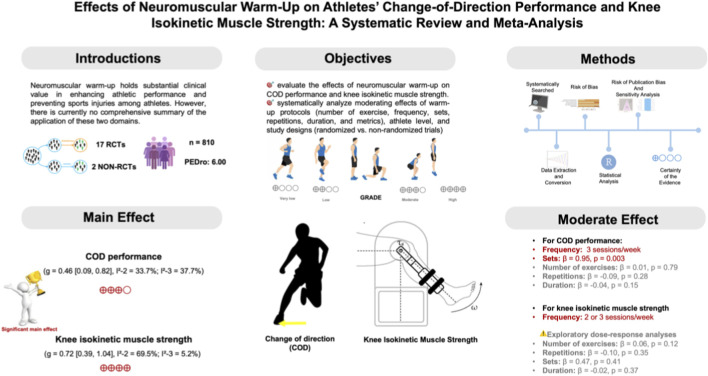

## 1 Introduction

Change of Direction (COD) performance is considered an essential quality for successful participation in many team and individual sports, which is a complex, multi-factorial quality (Brughelli et al., 2008). From a biomechanical perspective, factors such as shorter ground contact time, higher approach and exit velocities, greater braking and propulsive forces, a larger trunk inclination angle, a lower center-of-mass height, and increased moments and power at the hip, knee, and ankle contribute to faster COD completion times (Singh et al., 2025). Furthermore, high multi-planar and multi-action muscle force capacities are crucial for effective COD performance to facilitate frequent and large mechanical adjustments to control center of mass during various directional changes (Dos'Santos et al., 2017). Spiteri et al. (2014) previously showed that athletes must possess sufficient eccentric, concentric, dynamic, and isokinetic strength to change direction rapidly, thereby increasing the magnitude of force and impulse generation throughout the movement. Thus, well-developed muscle strength provides the necessary force output foundation for executing rapid deceleration, reorientation, and acceleration within sport-specific movements. These physical qualities are particularly important in sports that require frequent sprinting, pivoting, and sudden changes in movement direction (Dos'Santos et al., 2017).

Warm-up enhances athletic performance through various temperature-related mechanisms such as increased muscle temperature, improved blood flow, enhanced oxygen delivery, neural activation, and cardiovascular readiness (Bishop, 2003; Wilson et al., 2025), as well as non-temperature-related factors, most notably the phenomena encompassed by post-activation performance enhancement, which integrates neuromuscular potentiation, psychological readiness, and altered force-time characteristics (Wilson, 2020). Studies have shown that more than half (52%) of musculoskeletal injuries actually occur during daily training sessions (Ko et al., 2024), further highlighting the importance of implementing adequate warm-up routines in regular -practice (Chard and Lachmann, 1987). Warm-up strategies can generally be classified into passive and active methods (Bishop, 2003). Studies have shown that active warm-up protocols (e.g., jogging, dynamic stretching, and sport-specific drills) tend to produce more significant performance improvements compared to passive warm-up (e.g., heating pads or external warming methods) (effect size = 0.27 vs. 0.08) (Wilson et al., 2025).

Within the category of active warm-ups, neuromuscular warm-ups as a structured protocol containing at least three of the following exercise types: resistance, dynamic stability, core strength, plyometrics, and agility (Herman et al., 2012). It focus on the ‘synergistic activation of the neuromuscular-skeletal system. By integrating dynamic balance training (e.g., single-leg stance), safe landing technique correction (e.g., buffer movement control), postural stability enhancement (e.g., trunk tilt angle adjustment), and reactive force-generation exercises (e.g., rapid change-of-direction initiation), they simultaneously achieve the dual goals of ‘improving athletic performance’ and ‘reducing injury risk (Fernandez-Fernandez et al., 2020; Paravlic et al., 2024; Pasanen et al., 2009). A multi-population study by Herman et al. (2012) confirmed that this type of warm-up can reduce the incidence of lower limb injuries by 30%–50% in young amateur athletes, female athletes, and military personnel—mechanistically linked to improved activation timing of muscles around the knee joint (e.g., synergistic contraction of hamstrings and quadriceps).”

Knee isokinetic muscle strength, the gold standard for lower limb strength assessment (van Dyk et al., 2016), parameters measured by isokinetic dynamometers [such as peak torque and hamstring-to-quadriceps (H:Q) ratios] are core indicators for objectively evaluating muscle strength (Parraca et al., 2022; Perrin et al., 1987; Ruas et al., 2019). Research indicates that lower limb muscle strength is closely related to COD ability (Thomas et al., 2018), and deficits in hamstring and quadriceps isokinetic strength are considered weak risk factors for hamstring strain injuries (van Dyk et al., 2016). Thus, improvements in COD performance and knee isokinetic strength are regarded as critical indirect biomechanical and neuromuscular surrogates for injury risk reduction, rather than direct injury endpoints. Nevertheless, whether there exists a clear dose-response relationship between the specific design of neuromuscular warm-up protocols (such as content, frequency, load, and duration) and COD performance or knee isokinetic muscle strength indicators remains a subject for further in-depth research.

Current systematic reviews and meta-analyses on neuromuscular warm-up primarily focus on two main outcomes, injury risk reduction and athletic performance (Emery et al., 2015; Liu et al., 2021; Muller et al., 2023; Steib et al., 2017; Wang P. et al., 2024). Nevertheless, there are significant methodological and reporting limitations in these studies. From a methodological perspective, previous research has several shortcomings: Firstly, the criteria for adopting correlation coefficients are not clearly defined. Fourth, Secondly, traditional two-level meta-analysis models have not sufficiently considered the multilevel variability characteristics within and between studies (Cheung, 2014). Thirdly, there has been inadequate handling of outliers during data analysis (effect sizes greater than 3) (Kadlec et al., 2023). Fourth, the literature screening process generally lacks the application of snowball sampling, leading to the potential omission of important grey literature (Mahood et al., 2014). In terms of result interpretation, there is a common issue of insufficient analysis of moderating variables, specifically: 1) inadequate exploration of the dose-response relationship regarding training parameters (such as warm-up content, number of sets, and repetitions); 2) a lack of systematic analysis of differences in responses between different levels of athletes (trained vs. highly trained vs. elites); and 3) insufficient assessment of the potential biases that may arise from study design characteristics (such as randomized vs. non-randomized designs). As a result, there is currently a lack of systematic evaluations regarding the dose-response relationship of neuromuscular warm-up. This knowledge gap affects coaches’ ability to develop warm-up protocols based on reliable evidence.

To address these research gaps, this study will employ the following strategies: Firstly, a robust methodological screening process will be used to include relevant literature, and a three-level meta-analytic model will be implemented to investigate the chronic effects of neuromuscular warm-up implemented as repeated protocols across multiple weeks on COD performance and knee isokinetic muscle strength. Additionally, dose-response and moderator analyses will be conducted. Specifically, the effects of quantifiable prescription parameters (i.e., “dose” such as repetitions, sets, and duration) will be primarily examined via meta-regression of continuous variables, supplemented by subgroup analysis for categorical variables (such as athlete level and study design).

## 2 Materials and methods

The Preferred Reporting Items performed this systematic review for Systematic Reviews and Meta-Analyses (PRISMA) guidelines (Page et al., 2021). The completed PRISMA 2020 checklist is available in Supplementary Material S1. Additionally, this review has been registered in the PROSPERO database under the identifier CRD420251046324.

### 2.1 Information sources

Database searches were conducted in PubMed, Web of Science (Core Collection), Embase, and Scopus. For inclusion in this review, publications had to be full-text articles with no restrictions on publication date or sample. Articles were included if the title and abstract were available in English. Three systematic snowballing searches were applied: 1) checking the reference lists of included articles; 2) reviewing articles that cited the included articles; 3) exploring “similar articles” or “find similar” (PubMed, Embase). The searches were conducted from the earliest record upto 5 May 2025, and updated on 15 May 2025.

### 2.2 Search strategy

The search strategy was developed based on a previous review of similar topic (Concha-Cisternas et al., 2023; Steib et al., 2017). The following syntax was used: (“neuromuscular train*” OR “proprioceptive train*” OR “sensorimotor train*” OR “balance train*” OR “coordination train*” OR “plyometric train*” OR “agility train*” OR “functional train*” OR prehabilitation OR “neuromuscular warm-up” OR “dynamic warm-up” OR “pre-activity warm-up” OR “injury prevention warm-up” OR “FIFA 11+”) AND (performance OR “physical fitness” OR strength OR balance OR agility OR endurance OR jump* OR sprint* OR “change of direction” OR cod OR “postural control” OR “muscle activation” OR “reaction time” OR “injury prevention” OR “injury incidence”) NOT (animal OR mice OR rat). Additionally, searches of PROSPERO and the Cochrane Database of Systematic Reviews were conducted to determine whether protocols for related systematic reviews had already been published. The search form and corresponding results for other databases can be found in Table 1.

**TABLE 1:** Search results.

Data	Query	Results
PUBMED	((“neuromuscular train*” [Title/Abstract] OR “proprioceptive train*” [Title/Abstract] OR “sensorimotor train*” [Title/Abstract] OR “balance train*” [Title/Abstract] OR “coordination train*” [Title/Abstract] OR “plyometric train*” [Title/Abstract] OR “agility train*” [Title/Abstract] OR “functional train*” [Title/Abstract] OR prehabilitation [Title/Abstract] OR “neuromuscular warm-up” [Title/Abstract] OR “dynamic warm-up” [Title/Abstract] OR “pre-activity warm-up” [Title/Abstract] OR “injury prevention warm-up” [Title/Abstract] OR “FIFA 11+” [Title/Abstract]) AND (performance [Title/Abstract] OR “physical fitness” [Title/Abstract] OR strength [Title/Abstract] OR balance [Title/Abstract] OR agility [Title/Abstract] OR endurance [Title/Abstract] OR jump*[Title/Abstract] OR sprint*[Title/Abstract] OR “change of direction” [Title/Abstract] OR COD [Title/Abstract] OR “postural control” [Title/Abstract] OR “muscle activation” [Title/Abstract] OR “reaction time” [Title/Abstract] OR “injury prevention” [Title/Abstract] OR “injury incidence” [Title/Abstract])) NOT (animal [Title/Abstract] OR mice [Title/Abstract] OR rat [Title/Abstract])	4,998
Web of science	((TS=(“neuromuscular train*” OR “proprioceptive train*” OR “sensorimotor train*” OR “balance train*” OR “coordination train*” OR “plyometric train*” OR “agility train*” OR “functional train*” OR prehabilitation OR “neuromuscular warm-up” OR “dynamic warm-up” OR “pre-activity warm-up” OR “injury prevention warm-up” OR “FIFA 11+”)) AND TS=(performance OR “physical fitness” OR strength OR balance OR agility OR endurance OR jump* OR sprint* OR “change of direction” OR COD OR “postural control” OR “muscle activation” OR “reaction time” OR “injury prevention” OR “injury incidence”)) NOT TS=(animal OR mice OR rat)	4,887
embase	(“neuromuscular train*” OR ‘proprioceptive train*’ OR ‘sensorimotor train*’ OR ‘balance train*’ OR ‘coordination train*’ OR ‘plyometric train*’ OR ‘agility train*’ OR ‘functional train*’ OR ‘prehabilitation’/exp OR prehabilitation OR ‘neuromuscular warm-up’ OR ‘dynamic warm-up’ OR ‘pre-activity warm-up’ OR ‘injury prevention warm-up’ OR ‘fifa 11+’) AND (performance:ab,ti OR ‘physical fitness’:ab,ti OR strength:ab,ti OR balance:ab,ti OR agility:ab,ti OR endurance:ab,ti OR jump*:ab,ti OR sprint*:ab,ti OR ‘change of direction’:ab,ti OR cod:ab,ti OR ‘postural control’:ab,ti OR ‘muscle activation’:ab,ti OR ‘reaction time’:ab,ti OR ‘injury prevention’:ab,ti OR ‘injury incidence’:ab,ti) NOT (animal:ab,ti OR mice:ab,ti OR ‘rat’:ab,ti)	7,136
Scopus	(TITLE-ABS-KEY (“neuromuscular train*” OR “proprioceptive train*” OR “sensorimotor train*” OR “balance train*” OR “coordination train*” OR “plyometric train*” OR “agility train*” OR “functional train*” OR prehabilitation OR “neuromuscular warm-up” OR “dynamic warm-up” OR “pre-activity warm-up” OR “injury prevention warm-up” OR “FIFA 11+”) AND TITLE-ABS-KEY (performance OR “physical fitness” OR strength OR balance OR agility OR endurance OR jump* OR sprint* OR “change of direction” OR cod OR “postural control” OR “muscle activation” OR “reaction time” OR “injury prevention” OR “injury incidence”) AND NOT TITLE-ABS-KEY (animal OR mice OR rat))	8,230

### 2.3 Selection process

Deduplication of retrieved records was performed manually by an independent reviewer (M.L.T.) using Zotero [version 6.0.37]. Subsequently, the deduplicated literature was exported and provided to two independent researchers (M.L.T. and C.W.Z.) for screening the titles and abstracts based on predefined inclusion and exclusion criteria. If consensus could not be reached, a third independent researcher (C.Y.W.) reviewed the article to determine its inclusion status. The two independent researchers reviewed the full texts of selected articles for final inclusion. Additionally, potential sources of relevant articles included references from previous systematic reviews on the topic and the expertise of the research team, who identified articles that may meet the inclusion criteria for this review but were not initially captured in the literature search.

### 2.4 Eligibility criteria

This systematic review applied predefined inclusion and exclusion criteria following the PICOS framework (Population, Intervention, Comparison, Outcome, Study Design). Inclusion criteria required the population to be healthy human athletes of any age or training status. Interventions involved the application of neuromuscular warm-up protocols with clearly defined parameters (e.g., duration, frequency, number of exercises) and a minimum duration of 2 weeks. Comparisons included control groups receiving alternative warm-up protocols matched in parameters to the experimental group. Study designs encompassed RCTs or non-RCTs, prioritizing methodological rigor while ensuring inclusivity of relevant evidence.

Eligible studies were required to report at least one outcome of related to COD performance or knee isokinetic muscle strength. COD performance can be quantified by key biomechanical variables [e.g., total completion time (s)] in the acceleration and deceleration phases, followed by acceleration in varying directions (Sheppard and Young, 2006). Knee isokinetic muscle strength includes concentric peak torque, eccentric peak torque, conventional H:Q ratios, and functional H:Q ratios. Studies focusing on molecular-level mechanisms of physiological processes were excluded. Only original research with a between-group controlled trial design, including both parallel and cross-grouping, whether randomized or not, was included. Acute studies, review articles, opinions/viewpoint articles, validation studies, books, and case studies were excluded from consideration.

### 2.5 Data extraction

Data extraction was conducted by two reviewers (M.L.T. and C.W.Z.), utilizing a customized extraction worksheet in Excel that was finalized prior to the full-text review. Reviewers independently extracted information on author(s)' details, study characteristics, athlete level, warm-up protocols, and metrics. Discrepancies were resolved through discussion between the two reviewers, and no third researcher was required for arbitration. If data were missing or presented only in graphical form, the authors were contacted to request the necessary information. If this was unsuccessful and data remained in graphical form, relevant data were extracted using WebPlotDigitizer 4.1 (https://automeris.io/WebPlotDigitizer) (Drevon et al., 2017). For each group, the mean, standard deviation (SD), and sample size were extracted pre- and post-intervention.

### 2.6 Risk of bias and quality of methods assessment

The risk of bias was assessed using the Cochrane Collaboration's Risk of Bias Tool 2 (Rob2) (Sterne et al., 2019), which evaluates random sequence generation, random allocation concealment, blinding of outcome assessment, incomplete outcome data, and selective outcome reporting. Disagreements were resolved through discussion whenever possible. If consensus could not be reached, a third reviewer acted as an arbitrator. For non-randomized studies, Cochrane's Risk of Bias In Non-Randomized Studies of Interventions (ROBINS-I) (Sterne et al., 2016) was used, assessing bias across seven domains: confounding, participant selection, intervention categorization, adherence to intended interventions, handling of missing data, outcome measurement, and selection of reported results. Additionally, the physiotherapy evidence database (PEDro) (de Morton, 2009) scale was used to assess the risk of bias and methodological quality of included studies, which includes 11 core dimensions (e.g., ‘random allocation,’ ‘allocation concealment,’ ‘blinded outcome assessment,’ ‘completeness of follow-up’), with the first dimension ('clear eligibility criteria') excluded from scoring. The total score ranges from 0 to 10 points. According to the scale criteria: scores ≥6 were classified as high-quality studies (low bias risk), 4–5 as moderate-quality (moderate bias risk), and ≤3 as low-quality (high bias risk).

### 2.7 Statistical analysis

#### 2.7.1 Data synthesis and effect measures

We extracted the mean, SD, and sample size reported for each group pre- and post-intervention. We pooled effects using pre- and post-intervention differences ( M±SD ) for each outcome indicator. The mean difference ( $M_{\text{change}}$ ) and SD of the change ( ${\text{SD}}_{\text{change}}$ ) were calculated using the following formulae (Becker, 1988; Morris, 2008; Morris and DeShon, 2002), the first step involved calculating the difference in means using Equation 1:

$$M_{\text{change}}=M_{\text{post}}-M_{\text{pre}}$$ (1)

where $M_{\text{change}}$ is the raw mean difference, $M_{\text{post}}$ is the reported mean post-intervention, and $M_{\text{pre}}$ is the reported mean pre-intervention (Cumpston et al., 2019).

Then the ${\text{SD}}_{\text{change}}$ is calculated using Equation 2 (Cumpston et al., 2019):

$${\text{SD}}_{\text{change}}=\sqrt{{\text{SD}}_{\text{pre}}^{2}+{\text{SD}}_{\text{post}}^{2}-\left(2\times r\times {\text{SD}}_{\text{pre}}\times {\text{SD}}_{\text{post}}\right)}$$ (2)

where ${\text{SD}}_{\text{change}}$ is the SD of the difference in means, ${\text{SD}}_{\text{pre}}$ is the SD from pre-intervention, is the SD from post-intervention, and ${\text{SD}}_{\text{post}}$ is the correlation coefficient. (Cumpston et al., 2019) Correlation coefficients for pre- and post-intervention were rarely reported in the included studies and were generally assumed to be *r* = 0.50, as suggested by the Cochrane Handbook (Cumpston et al., 2019). However, to ensure the robustness of our results to this assumption, we conducted a pre-analysis sensitivity test for each primary outcome (COD performance and knee isokinetic strength). We fitted the three-level meta-analysis model across a plausible range of r values (0.5, 0.6, 0.7, 0.8, 0.9). The r value that yielded the overall effect size estimate closest to the median of all tested estimates (i.e., demonstrating the smallest absolute deviation) was selected for the primary analysis. While a simpler alternative is to use pooled SD of baseline scores, both and pooled SD of baseline scores are recommended (Lakens, 2013), each with advantages depending on the research question (Dunlap et al., 1996; Gibbons et al., 1993; Morris, 2000; Morris and DeShon, 2002).

Considering the relatively small sample sizes in most neuromuscular warm-up studies, Hedge's *g* was used as the mean effect size point estimate in each analysis, using the following formula for Hedge’s g, shown in Equation 3 (Hedges and Olkin, 1985):

$$\text{Hedge}’s\;g=\frac{\left(\text{Neuromuscular}\;\text{warm}-\text{up}\;\left[M_{\text{change}}\right]-C\text{ontrol}\;\left[M_{\text{change}}\right]\right)}{{\text{SD}}_{\text{pooled}}}\times \left(1-\frac{3}{4\left(n_{1}+n_{2}-2\right)-1}\right)$$ (3)

where $M_{\text{change}}$ is the mean difference between the neuromuscular warm-up and control groups, *n*_1_ and *n*_2_ are the sample sizes of these 2 groups, and ${\text{SD}}_{\text{pooled}}$ is the pooled SD of the measurements (Hedges and Olkin, 1985). The specific formula for SD_pooled_ is shown in Equation 4:

$${\text{SD}}_{\text{pooled}}=\sqrt{\frac{\left(\left(n_{1}-1\right)\times {\text{SD}}_{1}^{2}+\left(n_{2}-1\right)\times {\text{SD}}_{2}^{2}\right)}{\left(n_{1}+n_{2}-2\right)}}$$ (4)

where $n_{1}$ and $n_{2}$ are the sample sizes of the 2 groups, ${\text{SD}}_{1}$ and ${\text{SD}}_{2}$ are the SDs of both groups. Hedge's *g* were classified as *trivial* (<0.2), *small* (0.2–0.5), *medium* (>0.5–0.8), and *large* (>0.8) (Cohen, 1988).

#### 2.7.2 Meta-analysis and heterogeneity

We first applied a traditional two-level meta-analysis based on a generic inverse-variance pooling method to pool Hedges' *g* and were conducted using the *meta* and *metafor* packages in the statistical software R (V.4.2.0) (Viechtbauer, 2010). For the two-level meta-analysis, we utilized the DerSimonian-Laird approach (DerSimonian and Kacker, 2007), which is a random-effects model accounting for potential heterogeneity across studies. This model assumes that effect sizes are derived from a distribution of true effects rather than from a single homogeneous population. Given the variation in study designs, warm-up protocols, and populations, the random-effects model incorporates heterogeneity (Cumpston et al., 2019) by assuming that the underlying effects follow a normal distribution, leading to a more accurate and appropriate estimation of the overall effect size.

When studies involve nested designs (e.g., multiple testing indicators within a single study) or multiple effect sizes (Kadlec et al., 2023), the correlation between effect sizes undermines the ‘independence assumption' of traditional two-level meta-analyses (Van den Noortgate et al., 2013)—selecting only one effect size leads to key data loss, while including all may overestimate statistical power (Assink and Wibbelink, 2016). To resolve this issue, this study referenced the three-level analytical framework by Pustejovsky and Tipton (2022): first, the correlation coefficient between effect sizes was calculated using the vcalc function to construct an approximate variance-covariance matrix V, which corrects for sample overlap effects; second, a three-level model (Level 1: sampling variance, Level 2: within-study effect size variance, Level 3: between-study variance) was fitted using the rma. mv function (Cheung, 2019). Cluster-robust inference was further combined with the robust function from the clubSandwich package to optimize the accuracy of parameter estimation in small-sample scenarios.

We calculated 95% confidence intervals (CIs) using the Knapp-Hartung adjustment (test = ‘knha’) with t-distributions for individual coefficients and F-distributions for omnibus tests. For multilevel models fitted with rma. mv, degrees of freedom were approximated viadfs = ‘contain’. Additionally, we computed the prediction interval (PI) for metrics with >5 included studies based on the t-distribution, which measures the treatment effect considering heterogeneity and provides useful additional information compared to the CI and used to estimate the range of the overall parameter and to account for the uncertainty of future observations (Spineli and Pandis, 2020), especially considering the use of a random-effects model (Borg et al., 2024; IntHout et al., 2016). The between-study variability (i.e., heterogeneity) of the intervention effects within each intervention comparison was assessed with I^2^ (Nakagawa et al., 2017), and the magnitude of the between-study variance (τ^2^) estimated using the generalized DerSimonian and Laird (DerSimonian and Laird, 1986) estimator and the Q-profile approach. Therefore, the main analysis reports I^2^ with the following interpretations: 0%–25%, might not be important; 25%–50%, may represent moderate heterogeneity; 50%–75%, may represent substantial heterogeneity; and 75%–100%, considerable heterogeneity (Cumpston et al., 2019). Additionally, the statistical power of the primary pooled effect was calculated, and the possibility of false negatives due to insufficient statistical power was considered. Statistical power calculations were performed using the *metameta* package (Quintana, 2023).

#### 2.7.3 Subgroup and meta-regression analysis

To explore sources of heterogeneity among studies and moderating factors, this study employed subgroup and meta-regression analysis, conducting statistical analyses on binary and continuous variables (Hopkins and Batterham, 2018). It is generally recommended to have at least 10 studies available for each meta-regression, with a minimum of five studies per group for subgroup analyses (Deeks et al., 2019; Ruppar, 2020). In the present review, meta-regression was therefore performed only for outcomes with k ≥ 10. For knee isokinetic strength (k = 6), the number of studies was insufficient for a reliable meta-regression. However, to maintain full transparency and to inform future research, exploratory meta-regression analyses for knee isokinetic strength are provided in Supplementary File S1, where they are explicitly labelled as hypothesis-generating and interpreted with caution due to the limited study pool. Additionally, the statistical power of each subgroup was calculated to prevent the possibility of false negatives due to insufficient statistical power (Quintana, 2023).

We conducted subgroup analyses of the warm-up protocols about frequency (two, three, and four times per week) and testing metrics [COD (Illinois Agility Test, 505 test, sport-specific COD tasks, T-test, square test, v-cut, generic COD tests, change of direction test) and Knee isokinetic muscle strength (concentric peak torque, eccentric peak torque, functional H:Q ratio, and traditional H:Q ratio)], athlete level (trained vs. highly trained vs. elite athletes), and study designs. We conducted meta-regression analysis of the neuromuscular warm-up protocols included the number of exercises of the neuromuscular warm-up (Lopes et al., 2019; Trajković et al., 2020), sets, repetitions (Sannicandro et al., 2023; Panagoulis et al., 2020), duration (Lopes et al., 2019; Trajković et al., 2020). Regarding the directionality of effect sizes, a negative g indicates that the control group is better in terms of improvements in athletic performance, while a positive g indicates that neuromuscular intervention is better.

#### 2.7.4 Risk of publication bias and sensitivity analysis

The contour-enhanced funnel plot (Peters et al., 2008), in conjunction with Egger's asymmetry test (Egger et al., 1997; Fernández-Castilla et al., 2021) was employed to assess publication bias (tests were only conducted when *k indicates the number of included studies* ≥ 10 (Sterne et al., 2011)), and the *p* > 0.05 was considered without risk of publication bias. Funnel plots and Egger's regression tests are primarily used to determine the symmetry of the overall effect size, either through subjective or quantitative measures, thereby assessing the risk of publication bias in the included studies.

Sensitivity analyses were conducted as a leave-one-out analysis, sequentially removing each study to assess whether any single study significantly influenced the overall pooled effect. As a sensitivity analysis, we used cluster-robust variance estimation methods (Hedges et al., 2010) with small-sample adjustments (Tipton and Pustejovsky, 2015) to adjust the within-study standard errors for correlations between effect sizes. If the results changed significantly, we applied these methods; otherwise, we retained the original model.

Additionally, in the sensitivity analysis for the selection of the correlation coefficient (r), we systematically examined the assumed correlation coefficient required to construct the covariance matrix for the effect sizes. Since the true value of this parameter is unknown, we tested five values within a plausible range of 0.5–0.9 (r = 0.5, 0.6, 0.7, 0.8, 0.9). For each value of r, the corresponding covariance matrix was constructed using the impute_covariance_matrix function, and a three-level random-effects model (rma.mv) was fitted. Based on the principle of statistical stability, we calculated the absolute deviation of each model's overall effect size estimate from the median of these estimates. The correlation coefficient corresponding to the smallest absolute deviation was ultimately selected as the standard parameter for the primary analysis, ensuring the robustness of the results to variations in the assumed correlation.

### 2.8 Certainty of the evidence

The risk of bias was considered in the interpretation of the results by applying the Grading of Recommendations Assessment, Development, and Evaluation (GRADE) methodology, which rates the certainty of evidence as “high”, “moderate”, “low” or “very low” (Schünemann et al., 2019). GRADE assessments were initially completed by one reviewer and reviewed by a second reviewer for verification.

## 3 Results

### 3.1 Studies retrieved

**FIGURE 1:**
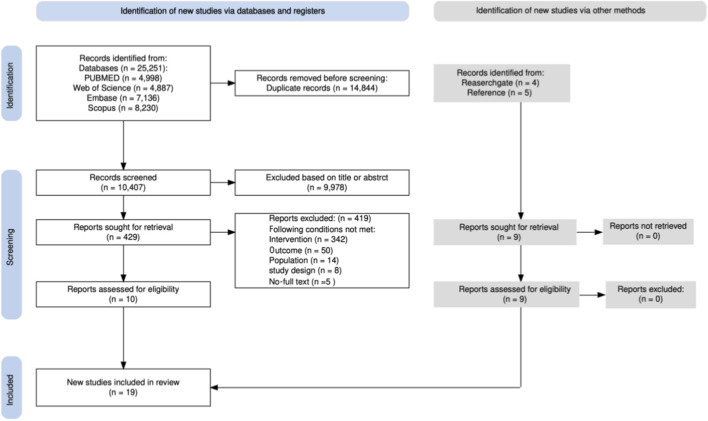
PRISMA flow diagram for included and excluded studies.

A flow diagram of the study selection process is presented in Figure 1. Overall, 25,251 studies were identified in the initial database search. After removing duplicates (n = 14,844), a total of 10,407 titles and abstracts were screened against the inclusion criteria, of which 9,978 studies were deemed irrelevant. A full-text review of the remaining 429 studies excluded an additional 419 studies for the following reasons: unable to contact the author (n = 5), not a neuromuscular warm-up (n = 342), inappropriate population (n = 14), outcome indicators not met (n = 50), and not a randomized controlled trial or controlled clinical trial (n = 8). After this process, 10 studies were eligible for inclusion. Following a snowball screening process (n = 9), a total of 19 studies were included in the meta-analysis (Arsenis, 2020; Asgari et al., 2022; Ayala et al., 2017; Abdolhamid et al., 2013; Daneshjoo et al., 2013; Fernandez-Fernandez et al., 2020; Hammami et al., 2023; Impellizzeri et al., 2013; Lindblom et al., 2012; Lopes et al., 2020; Nuhmani, 2021; Oliano et al., 2017; Panchal et al., 2025; Pardos-Mainer et al., 2019; Patti et al., 2022; Pomares-Noguera et al., 2018; Reis et al., 2013; Trajković et al., 2020; Zouhal et al., 2019).

### 3.2 Characteristics of included studies

A total of 19 studies (17 RCTs and 2 non-RCTs) involving 810 participants (male, n = 635; female, n = 175) were included. The sample size of individual studies ranged from 20 to 90 participants, with mean ages spanning from 11.02 to 28.03 years. All studies compared a neuromuscular warm-up intervention against a control condition, which primarily consisted of a regular warm-up (16 studies), with two studies using a dynamic warm-up protocol. In terms of athlete level, one study involved elite athletes, nine studies involved highly trained athletes, and nine studies involved trained athletes. Additional details are provided in Table 2

**TABLE 2:** The characteristics of the included studies.

First author, year	Characteristic	Design	Sample size	Age (yr)	Warm-up durations (weeks)	Frequency (times per week)	Control type
Arsenis (2020)	Highly trained	RCT	32	19.05 ± 0.83	8	3	Regular warm-up
Asgari et al. (2022)	Trained	RCT	90	16.88 ± 0.66	16	3	Regular warm-up
Ayala et al. (2017)	Highly trained	RCT	41	16.80 ± 0.70	4	3	Regular warm-up
Daneshjoo et al. (2013)	Highly trained	RCT	36	18.90 ± 1.40	8	3	Regular warm-up
Daneshjoo et al. (2013)	Highly trained	RCT	36	18.90 ± 1.40	8	3	Regular warm-up
Fernandez-Fernandez et al. (2020)	Highly trained	RCT	29	15.09 ± 1.16	8	3	Dynamic warm-up
Hammami et al. (2023)	Highly trained	RCT	24	15.55 ± 0.71	8	2	Regular warm-up
Impellizzeri et al. (2013)	Trained	RCT	81	23.45 ± 3.73	9	3	Regular warm-up
Lindblom et al. (2012)	Highly trained	RCT	41	14.20 ± 0.89	11	2	Regular warm-up
Lopes et al. (2020)	Trained	RCT	71	26.52 ± 5.09	10	2	Regular warm-up
Nuhmani (2021)	Trained	RCT	48	20.15 ± 2.19	12	3	Regular warm-up
Oliano et al. (2017)	Trained	RCT	21	13.10 ± 0.88	12	2	Regular warm-up
Panchal et al. (2025)	Trained	RCT	80	28.03 ± 6.80	8	4	Regular warm-up
Pardos-Mainer et al. (2019)	Highly trained	RCT	36	12.70 ± 0.60	10	2	Regular warm-up
Patti et al. (2022)	Highly trained	non-RCT	29	26.51 ± 6.95	5	2	Dynamic warm-up
Pomares-Noguera et al. (2018)	Trained	RCT	23	11.80 ± 0.30	4	2	Regular warm-up
Reis et al. (2013)	Trained	RCT	36	17.30 ± 0.70	12	2	Regular warm-up
Trajković et al. (2020)	Trained	non-RCT	36	11.02 ± 0.80	4	3	Regular warm-up
Zouhal et al. (2019)	elite	RCT	20	17.25 ± 0.72	6	2	Regular warm-up

RCT, randomized controlled trial; NON-RCT, randomized controlled trial; yr, years.

### 3.3 Primary analysis

**FIGURE 2:**
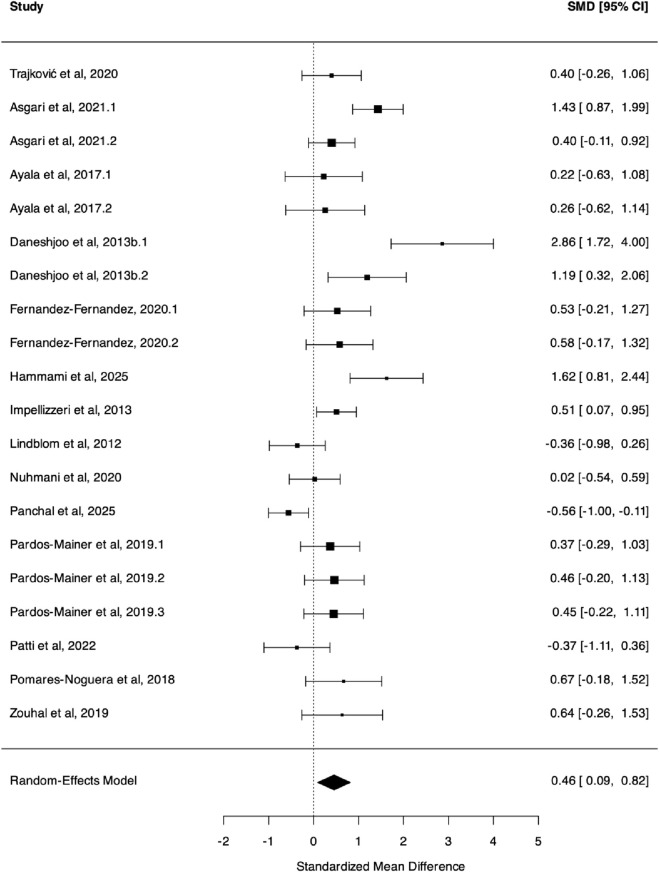
Three level meta analysis on change of direction performance. Note: Hedges'g, the effect size indicators used in the pooled; 95% CI, 95% confidence interval; COD performance” is measured by the time (typically in seconds) taken to complete a change-of-direction test (e.g., Illinois Agility Test, 505 test, T-test); A negative Hedges' g value indicates that the control group performed better (i.e., had a faster completion time) than the neuromuscular warm-up group.

Regarding COD performance, the meta-analysis found a significant improvement for neuromuscular warm-up compared to control (*k* = 14, *g* = 0.46, 95% CI [0.09, 0.82], *I* ^*2*^ *-2* = 33.7%; *I* ^*2*^ *-3* = 37.3%, *P* = 0.001, PI [-0.83, 1.74], Moderate GRADE, Figure 2).

**FIGURE 3:**
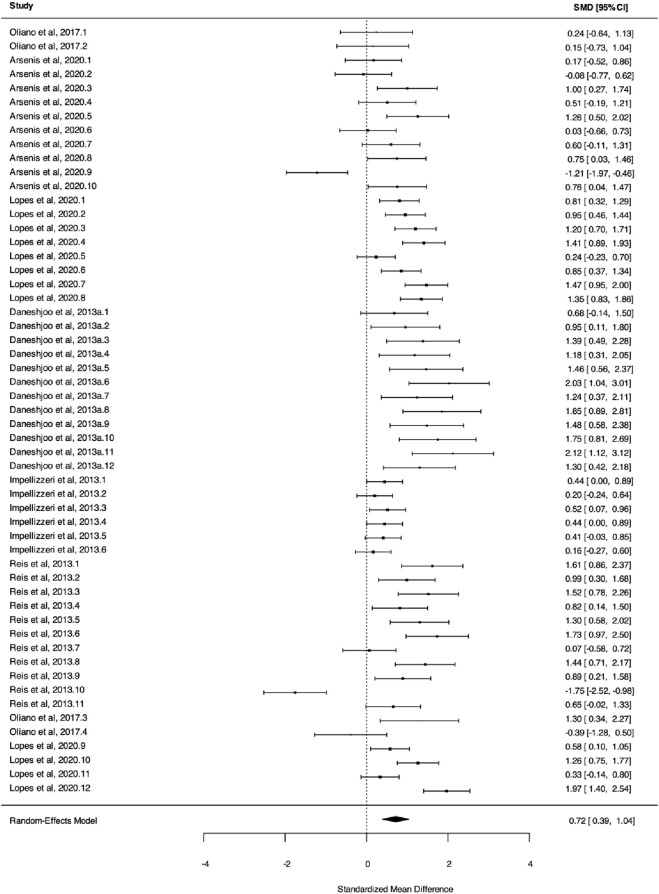
Three level meta analysis on knee isokinetic muscle strength. Note: Hedges'g, the effect size indicators used in the pooled; 95%CI, 95% confidence interval; knee isokinetic muscle strength” is measured by the [e.g., concentric peak torque, eccentric peak torque, functional hamstring-to-quadriceps (H:Q) ratio, and traditional H:Q ratio]; A negative Hedges' g value indicates a more favorable outcome for the control group (e.g., higher peak torque, more favorable hamstring-to-quadriceps ratio).

Regarding knee isokinetic muscle strength, the meta-analysis found a significant improvement for neuromuscular warm-up compared to control (*k* = 6, *g* = 0.72, 95% CI [0.39, 1.04], *I* ^*2*^ *-2* = 69.5%; *I* ^*2*^ *-3* = 5.2%, *P* < 0.001, PI [-0.58, 2.01], High GRADE, Figure 3).

**FIGURE 4:**
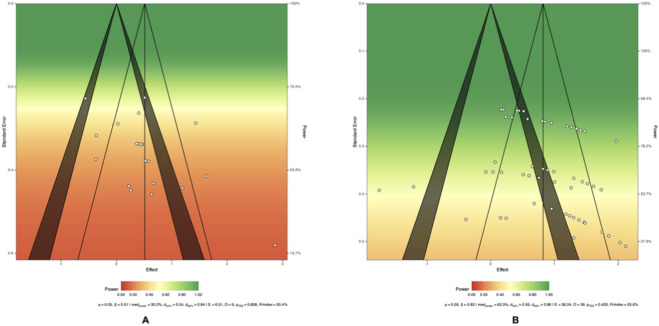
Power visualization. **(A)** COD Performance. **(B)** Knee Isokinetic Muscle Strength. Note: The vertical solid line represents the pooled effect size, and the vertical dash line represents the adjusted pooled effect size. Significance contours at 0.05 and 0.01 levels are noted by the shaded area. manpower indicates the median power of all included effect sizes. d33% and d66% indicate the true effect sizes necessary for achieving 33% and 66% levels of median power. E, O, and PTES show the results of a test of excess significance. R-index denotes the expected replicability of findings.

A visual plot of statistical power for the pooled results for all outcomes is provided in Figure 4.

### 3.4 Moderator analysis

We conducted moderator analysis to explore the modifying effects of neuromuscular warm-up protocol, athlete level, and study design on COD performance and knee isokinetic muscle strength.

#### 3.4.1 Potential moderators of COD performance

Significant moderating effects were found for the warm-up protocols, participants' characteristics, and study design on COD performance (*p* < 0.05) (Table 3).

Regarding neuromuscular warm-up protocols, three times per week (g = 0.68, *p* < 0.01) showed significantly greater improvements in COD performance compared to two times per week (g = 0.19, *p* = 0.19) and four times per week (g = −0.56, *p* = NA).

**FIGURE 5:**
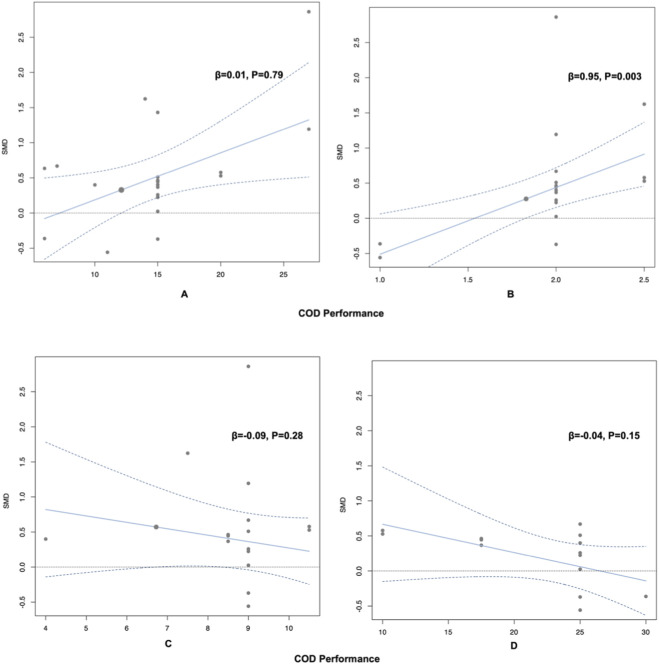
Meta-regression analysis based on neuromuscular warm-up protocols. **(A)** Exercises. **(B)** Sets. **(C)** Repetitions. **(D)** Durations. Note: The horizontal axis represents the neuromuscular warm-up protocols (number of exercise, sets, repetitions, durations), and the vertical axis represents the change in COD performance from pre to post intervention. Each bubble corresponds to one effect size.

Linear regression analyses revealed no significant linear relationship between COD performance improvements and neuromuscular warm-up excercise (β = 0.01, p = 0.79), repetitions (β = −0.09, p = 0.28), or the duration per exercise or set (i.e., the time spent holding or performing a single movement, such as for dynamic stability or core strength tasks) (β = −0.04, p = 0.15). However, a significant positive linear relationship was observed with sets (β = 0.95, p = 0.003) (Figure 5).

**TABLE 3:** Subgroup analyses of COD performance.

Subgroup	NWU	CON	Hedges' g [95%CI]	P	I^2^	Power
NWU protocol						
Frequency						
Two times per week	137	124	0.19 [-0.09; 0.48]	0.19	25%	42.20%
Three times per week	216	216	0.68 [ 0.31; 1.06]	<0.01	67%	57.80%
Four times per week	40	40	−0.56 [-1.00, −0.11]	NA	NA	6.1%
Testing metrics						
Illinois agility test	159	150	0.72 [ 0.18; 1.27]	<0.01	77%	44.70%
505 test	28	30	0.55 [ 0.03; 1.08]	0.04	0%	10.10%
Sport-specific COD tasks	10	10	0.64 [-0.27; 1.54]	NA	NA	4.20%
T-test	79	79	0.12 [-0.38; 0.62]	0.64	56%	18.40%
Square test	20	20	0.00 [-0.62; 0.62]	NA	NA	5.80%
V-cut	19	17	0.37 [-0.29; 1.03]	NA	NA	5.60%
Generic COD tests	38	34	0.45 [-0.01; 0.92]	0.06	0%	11.10%
Athlete level						
Trained athletes	197	191	0.39 [-0.09, 0.88]	0.11	81%	39.00%
Highly trained athletes	182	174	0.58 [ 0.16, 0.99]	<0.01	71%	56.70%
Elite athletes	10	10	0.64 [-0.27, 1.54]	NA	NA	4.3%
Study design						
RCTs	320	308	0.53 [ 0.26; 0.80]	<0.01	62%	89.30%
non-RCTs	33	32	0.33 [-0.72; 0.79]	0.93	57%	10.70%

COD, change of direction; NWU, Neuromuscular warm-up; CON, control; Hedges' g, the effect size indicators used in the pooled; 95%CI, 95% confidence interval; P-value, statistically significant P values for pooled results; I^2^, quantitative indicators of heterogeneity; Power, statistical power for pooled effect size; RCT, randomized controlled trial; NON-RCT, randomized controlled trial.

Testing metrics with the Illinois Agility Test (g = 0.72, *p* < 0.01) showed significantly greater improvements in COD performance compared to the 505 test (g = 0.55, *p* = 0.04), sport-specific COD tasks (g = 0.64, *p* = NA), T-test (g = 0.12, *p* = 0.64), square test (g = 0.00, *p* = NA), v-cut (g = 0.37, *p* = NA), and generic COD tests (g = 0.45, *p* = 0.06).

Regarding athlete level, highly trained athletes (g = 0.58, *p* < 0.01) showed greater improvements in COD performance compared to elite (g = 0.64, *p* = NA) and trained athletes (g = 0.39, *p* = 0.11).

Regarding study design, RCTs (g = 0.53, *p* < 0.01) showed significantly greater improvements in COD performance compared to non-RCTs (g = 0.03, *p* = 0.93).

#### 3.4.2 Potential moderators of knee isokinetic muscle strength

Significant moderating effects were found for the warm-up protocols, participants' characteristics, and study design on knee isokinetic muscle strength (*p* < 0.05) (Table 4).

**TABLE 4:** Subgroup analyses of knee isokinetic muscle strength.

Subgroup	NWU	CON	Hedges' g [95%CI]	P	I^2^	Power
NWU protocol						
Frequency						
Two times per week	674	658	0.87 [ 0.59; 1.14]	<0.01	79%	50.80%
Three times per week	556	538	0.75 [ 0.51; 1.02]	<0.01	69%	49.20%
Testing metrics						
Concentric peak torque	824	798	0.86 [ 0.66; 1.07]	<0.01	69%	68.40%
eccentric peak torque	134	128	0.70 [ 0.26; 1.15]	<0.01	64%	9.60%
Functional hamstring-to-quadriceps ratio	110	104	1.34 [ 0.81; 1.87]	<0.01	66%	7.70%
Traditional hamstring-to-quadriceps ratio	162	166	0.34 [-0.39; 1.07]	0.29	86%	14.40%
Athlete level						
Trained athletes	926	892	0.77 [ 0.55, 0.98]	<0.01	78%	64.10%
Highly trained athletes	304	304	0.92 [ 0.59, 1.24]	<0.01	71%	35.90%

NWU: Neuromuscular warm-up; CON, control; Hedges' g, the effect size indicators used in the pooled; 95%CI, 95% confidence interval; P-value, statistically significant P values for pooled results; I^2^, quantitative indicators of heterogeneity; Power, statistical power for pooled effect size; RCT, randomized controlled trial; NON-RCT, randomized controlled trial.

Regarding neuromuscular warm-up protocols, both two times per week (g = 0.87, *p* < 0.01) and three times per week (g = 0.75, *p* < 0.01) showed significant improvements in knee isokinetic muscle strength.

Due to the limited number of studies (k = 6), dose-response meta-regression for knee isokinetic strength was not included in the main analysis. Exploratory meta-regression examining the effects of exercise number, sets, repetitions, and duration is provided in Supplementary File S1. Linear regression analyses revealed no significant linear relationship between COD performance improvements and neuromuscular warm-up excercise (β = 0.06, p = 0.12), sets (β = 0.47, p = 0.41), repetitions (β = −0.10, p = 0.35), or the duration per exercise or set (i.e., the time spent holding or performing a single movement, such as for dynamic stability or core strength tasks) (β = −0.02, p = 0.37). These results should be regarded as hypothesis-generating only (Figure 5).

Regarding athlete level highly trained athletes (g = 0.92, *p* < 0.01) showed greater improvements in knee isokinetic muscle strength compared to trained athletes (g = 0.77, *p* < 0.01).

Regarding study design, all included studies on knee isokinetic muscle strength were RCTs (g = 0.82, *p* < 0.01), which precluded subgroup comparisons.

### 3.5 Risk of bias and quality of methods

**FIGURE 6:**
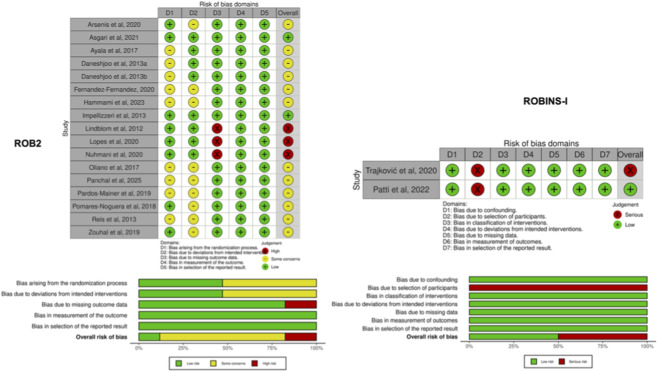
Risk of bias for the included studies.

The risk of bias for each study is presented in Figure 6. Regarding the seventeen RCTs, 52.9% of the studies did not disclose specific details about their randomization methods and allocation concealment, leading to an assessment of “some concerns” regarding the randomization process. Furthermore, due to dropout rates exceeding 85% in 17.6% of the studies, these were also flagged with “high risk.” Overall, most studies exhibited “some concerns” regarding bias. As for the two non-RCTs, both were assessed as having “serious” bias because the subjects, assessors, and data analysts did not implement blinding with respect to group information.

**FIGURE 7:**
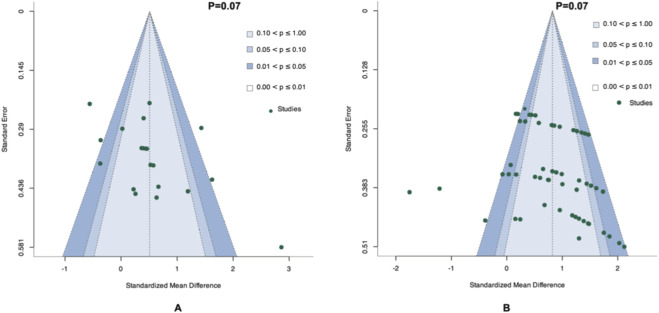
Funnel plot of included studies. **(A)** COD Performance. **(B)** Knee Isokinetic Muscle Strength.

A funnel plot combined with Egger's test was used to investigate the risk of publication bias for the effects of the included studies on COD performance and knee isokinetic muscle strength (Figure 7). No significant risk of publication bias was found for COD performance (p = 0.07) and knee isokinetic muscle strength (p = 0.07).

Among the 18 studies finally included in this research, 9 had PEDro scores ≥6 (accounting for 50%), and 9 had scores of 4–5, with an average score of 6.00. All studies met key dimensions such as ‘baseline balance between groups’ and ‘statistical comparison of outcome indicators,’ confirming that the overall methodological quality meets the requirements of systematic reviews. The average PEDro score of all studies was 6.00 (Table 5).

**TABLE 5:** Methodological quality assessment [PEDro].

First author, year	D1	D2	D3	D4	D5	D6	D7	D8	D9	D10	D11	Total
Arsenis (2020)	1	1	1	1	0	0	0	1	1	1	1	7
Asgari et al. (2022)	1	1	1	1	0	1	1	1	1	1	1	9
Ayala et al. (2017)	1	0	0	0	0	0	1	1	1	1	1	5
Daneshjoo et al. (2013)	1	0	0	1	0	0	1	1	1	1	1	6
Daneshjoo et al. (2013)	1	0	0	1	0	0	1	1	1	1	1	6
Fernandez-Fernandez et al. (2020)	1	0	0	1	0	0	0	1	1	1	1	5
Hammami et al. (2023)	1	0	0	1	0	0	0	1	1	1	1	5
Impellizzeri et al. (2013)	1	1	1	1	0	0	1	1	1	1	1	8
Lindblom et al. (2012)	1	1	1	1	1	1	1	0	1	1	1	9
Lopes et al. (2020)	1	1	1	1	0	1	0	0	0	1	1	6
Panchal et al. (2025)	1	0	0	1	0	0	0	1	0	1	1	4
Nuhmani (2021)	1	1	1	1	0	1	0	0	0	1	1	6
Oliano et al. (2017)	1	0	0	1	0	0	0	1	1	1	1	5
Pardos-Mainer et al. (2019)	1	0	0	1	0	0	0	1	1	1	1	5
Patti et al. (2022)	1	0	0	1	0	0	0	1	1	1	1	5
Pomares-Noguera et al. (2018)	1	1	1	1	0	0	0	1	1	1	1	7
Reis et al. (2013)	1	0	0	1	0	0	0	1	1	1	1	5
Trajković et al. (2020)	1	0	0	1	0	0	0	1	1	1	1	5
Zouhal et al. (2019)	1	1	0	1	0	0	0	1	1	1	1	6

Studies scoring ≥6 are considered high quality, those scoring 4-5 are considered moderate quality, and those scoring ≤3 are considered low quality.

1 eligibility criteria were specified (not included in the total score).

2 subjects were randomly allocated to groups (in a crossover study, subjects were randomly allocated an order in which treatments were received).

3 allocation was concealed.

4 the groups were similar at baseline regarding the most important prognostic indicators.

5 there was blinding of all subjects.

6 there was blinding of all therapists who administered the therapy.

7 there was blinding of all assessors who measured at least one key outcome.

8 measures of at least one key outcome were obtained from more than 85% of the subjects initially allocated to groups.

9 all subjects for whom outcome measures were available received the treatment or control condition as allocated or, where this was not the case, data for at least one key outcome was analysed by “intention to treat”.

10 the results of between-group statistical comparisons are reported for at least one key outcome.

11 the study provides both point measures and measures of variability for at least one key outcome.

### 3.6 Sensitivity analysis

**FIGURE 8:**
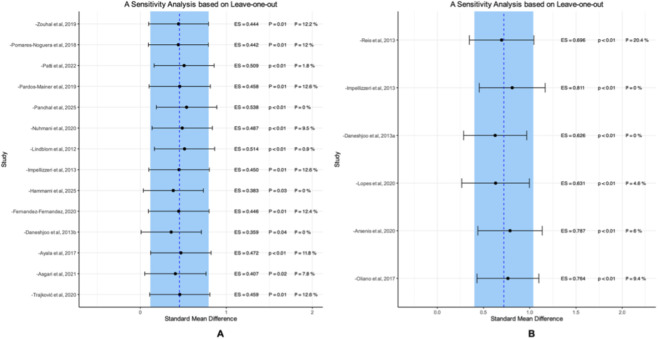
Sensitivity analysis based on leave-one-out. **(A)** COD Performance. **(B)** Knee Isokinetic Muscle Strength.

Results from the sensitivity analyses using a leave-one-out method is provided in Figure 8. The results indicated that excluding any single study did not significantly impact the overall pooled outcome, indicating the robustness and reliability of our findings Based on the evidence set by Kadlec et al. (2023), we adopted a conservative strategy to exclude outliers in order to enhance the robustness of the results.

**FIGURE 9:**
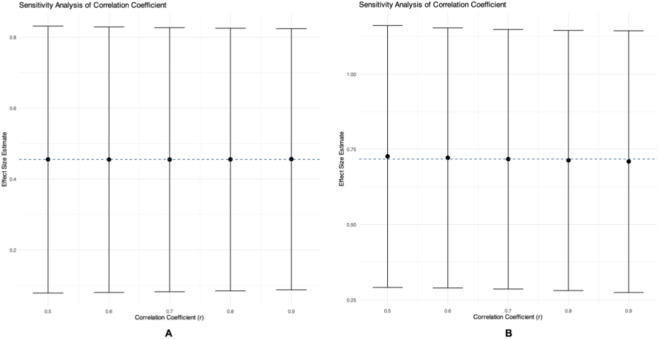
Sensitivity analysis based on Selection R. **(A)** COD Performance. **(B)** Knee Isokinetic Muscle Strength.

A sensitivity analysis was performed to assess the impact of the assumed pre-post correlation coefficient (r) on the pooled effect estimates. For COD performance, the overall effect size (g) remained highly consistent across all tested r values (0.5–0.9), ranging from 0.455 to 0.456. The model assuming r = 0.5 yielded the estimate closest to the median and was therefore selected for the primary analysis. For knee isokinetic strength, the estimates were also stable, ranging from 0.709 to 0.726 across the same range of r values, with the model assuming r = 0.7 providing the most central estimate. The robustness of the primary findings to variations in this statistical parameter is thereby confirmed. Detailed results of this sensitivity analysis are presented in Figure 9.

## 4 Discussions

This study employed a three-level meta-analysis to investigate the effects of neuromuscular warm-up on athletes' COD performance and knee isokinetic muscle strength, addressing the limitations of previous meta-analyses on neuromuscular warm-up. The results showed that neuromuscular warm-up significantly improved athletes' COD performance and knee isokinetic muscle strength. Furthermore, several protocol parameters athlete level, and study designand showed significant associations with the magnitude of improvement. These findings contribute to understanding the extent of neuromuscular warm-up's impact, providing evidence-based references for designing neuromuscular warm-up protocols.

### 4.1 Primary effect

COD is a fundamental physical attribute in team (Paul et al., 2016; Sheppard and Young, 2006) and individual sports (Fernandez-Fernandez et al., 2014). The main effect results show that neuromuscular warm-up significantly increased COD performance (g = 0.46) in athletes. Factors influencing COD performance include anthropometric characteristics, technical skills, straight-line sprint speed, and lower-body strength (Young et al., 2002). Neuromuscular warm-up typically consists of aerobic, balance, strength, and agility exercises (Herman et al., 2012). Therefore, compared to a control group, this warm-up method may strengthen the COD technique specifically through the inclusion of agility exercises.

In the studies we included Our analysis highlighted that testing indicators for knee isokinetic muscle strength primarily consisted of peak torque and the H:Q ratio. Peak torque, a central metric in isokinetic strength testing, reflects the maximum force-generating capacity of lower limb joints at specific angles (Osawa et al., 2018). This capacity is critically dependent on, and fundamentally underpins, superior athletic performance in tasks requiring rapid direction changes, abrupt decelerations, and explosive accelerations (Hung et al., 2020). The strength of the agonist to antagonist muscles using the H:Q ratio has been helpful in the identification of injury risk and mitigation of strength imbalances to reduce injuries to the lower extremity (Lutz et al., 2022). The main effect results show that neuromuscular warm-up significantly increased knee isokinetic muscle strength (g = 0.72) in athletes. This further confirms the effectiveness of neuromuscular warm-up in enhancing knee joint muscle strength and thereby preventing knee injuries (Herman et al., 2012).

### 4.2 Moderator effect

Subgroup and meta-regression analyses were conducted to explore potential associations between program characteristics, athlete level, study design, and the observed improvements in COD performance and knee isokinetic muscle strength. It is crucial to interpret these findings with caution. Several subgroup comparisons were limited by a small number of studies, and the results should be viewed as generating hypotheses for future research rather than as definitive prescriptive evidence.

Regarding warm-up frequency, performing neuromuscular warm-up three times per week can significantly improve COD performance and knee isokinetic muscle strength. Similarly, we observed a similar trend in our studies to other meta-analyses in the same area (Akbar et al., 2022; Liu et al., 2021). We found that the warm-up frequencies in the included studies were limited to two or three times per week, for a frequency of four times per week was based on a single study (g = −0.56, p = NA) and should be interpreted as a preliminary observation only. We conducted subgroup analysis for intervention frequency primarily because the evidence was concentrated within two categories (two and three times per week), with only a single study investigating a frequency of four times per week. Similarly, to preliminarily explore the potential influence of control group type, we conducted an exploratory subgroup analysis. For knee isokinetic muscle strength, all comparators were regular warm-up. For COD performance, while most type were regular warm-up (g = 0.55 [0.22, 0.89]), only two studies (three effect sizes) used an dynamic warm-up (g = 0.24 [-0.36, 0.85]). The very limited evidence for the latter group necessitates extremely cautious interpretation regarding comparator effects.

For COD performance, meta-regression indicated a significant positive linear dose-response relationship with the number of sets, suggesting that increasing the number of sets per exercise is associated with greater improvement. However, no significant linear relationships were found with other parameters, including the number of exercises, repetitions, or set duration.

For knee isokinetic muscle strength, the number of available studies (k = 6) was insufficient to perform a statistically stable dose-response meta-regression. To maintain full transparency and to inform future research, exploratory dose-response analyses for this outcome are provided in Supplementary File S1. These results should be regarded as hypothesis-generating only, given the limited study pool and consequent risk of unstable estimates. At present, the evidence base does not permit definitive conclusions regarding linear dose-response relationships between neuromuscular warm-up prescription parameters and improvements in knee isokinetic strength.

In the included studies, we found that the range of neuromuscular warm-up components varied from six to twenty-seven, with warm-up durations ranging from fifteen to 40 min. Since neuromuscular warm-ups include stretching, strengthening, balance exercises, and sport-specific agility training, it is possible that a more comprehensive warm-up program with more than fifteen components (with at least two sets for each component) can more effectively enhance muscle temperature and function (Wilson et al., 2025). Furthermore, for timed components (e.g., isometric holds), a set duration of 25 s or less is often recommended to ensure an adequate activation stimulus while avoiding premature fatigue (Afonso et al., 2024; Blazevich and Babault, 2019; Tomaras and MacIntosh, 2011). It is important to emphasize that these parameter ranges require flexible adaptation and optimization based on individual athlete characteristics, specific sport demands, and contextual factors.

Regarding warm-up testing metrics, Liu et al. (Liu et al., 2021) also observed a similar trend to our study, indicating that the Illinois test appears to be a sensitive indicator for detecting changes in COD performance. It should be noted, however, that the Illinois test requires the athlete to sprint 9.20 m on command, turn, return to the starting line, then weave through four markers and complete two additional 9.20-m sprints to finish the agility course. Due to its inclusion of longer sprints and multiple weaving turns, the test imposes energy system and neuromuscular demands that differ considerably from those required for short-duration, high-intensity directional changes typical of actual competition. Although this test may simulate dribbling scenarios in ball sports such as football (Hachana et al., 2013) —which is relevant given the predominance of ball sport athletes in our sample—its applicability for assessing pure COD capability in athletes from other sports remains uncertain. Therefore, we recommend that future studies adopt shorter-duration, higher-intensity COD tests to more accurately reflect the biomechanical and metabolic characteristics of sport-specific directional changes. Meanwhile, in exploring knee isokinetic muscle strength, we found that concentric and eccentric peak torque, as well as functional H:Q ratios, are sensitive indicators for detecting the effects of neuromuscular warm-up on changes in knee muscle strength. Peak torque represents the maximum force generated by a muscle during a single contraction and is a key indicator of muscle strength (Baltzopoulos and Brodie, 1989). The H:Q ratio serves as a sensitive clinical tool for predicting injury risk and monitoring knee joint integrity (Ruas et al., 2019). Currently, there are two categories of the H:Q ratio: Conventional and Functional. Conventional H:Q ratio was calculated as the concentric hamstring to concentric quadriceps peak torque. Functional ratios were calculated as the eccentric quadriceps to concentric hamstring peak torque and as the concentric quadriceps to eccentric hamstring torque (Guney et al., 2016). Therefore, we recommend using concentric and eccentric peak torque, along with functional H:Q ratios, to assess warm-up effects and monitor athletes' injury risks.

Regarding athlete level, a consistent trend was observed wherein highly trained athletes showed larger improvements in both COD performance and knee strength compared to their trained athletes. This difference may be attributed to the former group's superior physiological advantages—such as maximum power output, running economy, anaerobic threshold, and maximum oxygen uptake (Lorenz et al., 2013). And more refined movement mechanics, such as the ‘short ground contact time–high propulsive force’ pattern essential for efficient COD (Wang B. et al., 2024). It is plausible that these characteristics allow highly trained athletes to more effectively translate the neuromuscular activation from a warm-up into performance enhancement. The point estimate for elite athletes (derived from a single study) suggested a potentially large effect size; however, this finding is highly preliminary (p = NA) and requires replication in future research. Therefore, while athlete training level appears to be a significant moderator, with more trained individuals tending to benefit more, the current evidence is strongest for distinguishing between trained and highly trained cohorts, and any extrapolation to elite populations remains speculative.

Regarding study design, we found that the trends observed in RCTs (g = 0.53) were consistent with those in non-RCTs (g = 0.03). This further validates the conclusion by Shadish et al. (2008) that, under certain conditions, the results of non-RCTs can closely approach or even reach the results of RCTs. The decision to cautiously include non-RCTs in this study was grounded in multiple methodological considerations: while RCTs remain the gold standard for intervention research (Hariton and Locascio, 2018), to balance evidence integrity and rigor, we adhered to PRISMA guidelines (Page et al., 2021) and selected non-RCTs were assessed by the ROBINS-I tool (Sterne et al., 2016). Sensitivity analyses showed stable effect directions for physiological adaptations and athletic performance metrics when combining RCTs and non-RCTs data, with no significant fluctuations in heterogeneity, confirming the robustness of the evidence body. Through multidimensional quality controls, the complementary inclusion of non-RCTs not only expanded the evidence base but also did not compromise the reliability of the core conclusions.

Regarding quality of included studies, the average PEDro score was 6.00, with 12 of 19 studies scoring ≥6. When integrated with the Cochrane RoB 2 and ROBINS-I assessments, this indicates that while many studies met several key methodological criteria (e.g., baseline comparability, point estimates and variability reported), important concerns remain. Specifically, the RoB 2 tool revealed that a majority of RCTs raised ‘some concerns,’ primarily due to inadequate reporting of randomization and allocation concealment. Furthermore, the included non-RCTs were judged to have ‘serious’ risk of bias. Therefore, the overall body of evidence should be considered when interpreting the synthesized results.

### 4.3 Potential limitations

The following limitations should be acknowledged when interpreting the findings of this review. First, while the inclusion of healthy athletes across a spectrum of training levels enhances the generalizability of the results to athletic populations, the predominant focus on team-sport athletes limits the applicability of our conclusions to athletes in individual or cyclic sports. Future research should incorporate participants from a wider variety of sports disciplines to examine the context-specific effects of neuromuscular warm-up.

Second, the strength of conclusions drawn from moderator analyses is constrained by the limited number of studies within several subgroup categories. Specifically, for knee isokinetic muscle strength, the dose-response meta-regression was severely underpowered (k = 6) and has therefore been relegated to the Supplementary Materials as a highly exploratory analysis; these results should not be interpreted as evidence for or against dose-response relationships.

Finally, future research directions should instead focus on the aforementioned need for diversified samples and on conducting high-quality trials with detailed reporting of warm-up parameters to facilitate more precise dose-response analyses.

## 5 Conclusions

Neuromuscular warm-up significantly enhances COD performance and knee isokinetic muscle strength compared to the control group, moderated by warm-up protocols (frequency, sets, and metrics), athlete level, and study designs. A linear dose-response relationship was identified specifically for the number of sets in relation to COD improvements; however, no such relationships were observed for other prescription parameters or for knee isokinetic strength. The latter finding is based on an exploratory, underpowered analysis and requires confirmation in future research. These findings support the implementation of neuromuscular warm-up as an effective strategy for enhancing sport-specific agility and knee strength in athletic populations.

## Data Availability

The datasets presented in this study can be found in online repositories. The names of the repository/repositories and accession number(s) can be found in the article/Supplementary Material.

## References

[B1] Abdolhamid D. Mokhtar A. H. Rahnama N. Yusof A. (2013). Effects of the 11+ and harmoknee Warm-up programs on physical performance measures in professional soccer players. J. Sports Sci. and Med. 12 (3), 489–496. 24149156 PMC3772593

[B2] Afonso J. Brito J. Abade E. Rendeiro-Pinho G. Baptista I. Figueiredo P. (2024). Revisiting the ‘Whys’ and ‘Hows’ of the warm-up: are we asking the right questions? Sports Med. Auckl. 54 (1), 23–30. 10.1007/s40279-023-01908-y 37658965 PMC10798919

[B3] Akbar S. Soh K. G. Jazaily M. N. N. Bashir M. Cao S. (2022). Effects of neuromuscular training on athletes physical fitness in sports: a systematic review. Front. Physiology 13, 939042. 10.3389/fphys.2022.939042 PMC9540396 36213248

[B4] Arsenis S. Gioftsidou A. Ispirlidis I. Kyranoudis A. Pafis G. Malliou P. (2020). Effects of the FIFA 11+ injury prevention program on lower limb strength and balance. J. Phys. Educ. Sport 20 (02), 592–598. 10.7752/jpes.2020.02087

[B5] Asgari M. Alizadeh M. H. Shahrbanian S. Nolte K. Jaitner T. (2022). Effects of the FIFA 11+ and a modified warm-up programme on injury prevention and performance improvement among youth male football players. PLOS ONE Oliveira RFS 17 (10), e0275545. 10.1371/journal.pone.0275545 36264894 PMC9584367

[B6] Assink M. Wibbelink C. J. M. (2016). Fitting three-level meta-analytic models in R: a step-by-step tutorial. Tutorials Quantitative Methods Psychol. 12 (3), 154–174. 10.20982/tqmp.12.3.p154

[B7] Ayala F. Pomares-Noguera C. Robles-Palazón F. J. Del Pilar García-Vaquero M. Ruiz-Pérez I. Hernández-Sánchez S. (2017). Training effects of the FIFA 11+ and harmoknee on several neuromuscular parameters of physical performance measures. Int. Journal Sports Medicine 38 (4), 278–289. 10.1055/s-0042-121260 28192831

[B8] Baltzopoulos V. Brodie D. A. (1989). Isokinetic dynamometry. Applications and limitations. Sports Med. Auckl. 8 (2), 101–116. 10.2165/00007256-198908020-00003 2675256

[B9] Becker B. J. (1988). Synthesizing standardized mean-change measures. Br. J. Math. Stat. Psychol. 41 (2), 257–278. 10.1111/j.2044-8317.1988.tb00901.x

[B10] Bishop D. (2003). Warm up I: potential mechanisms and the effects of passive warm up on exercise performance. Sports Med. 33 (6), 439–454. 10.2165/00007256-200333060-00005 12744717

[B11] Blazevich A. J. Babault N. (2019). Post-activation potentiation *versus* post-activation performance enhancement in humans: historical perspective, underlying mechanisms, and current issues. Front. Physiology 10, 1359. 10.3389/fphys.2019.01359 31736781 PMC6838751

[B12] Borg D. N. Impellizzeri F. M. Borg S. J. Hutchins K. P. Stewart I. B. Jones T. (2024). Meta‐analysis prediction intervals are under reported in sport and exercise medicine. Scand. J. Med. and Sci. Sports 34 (3), e14603. 10.1111/sms.14603 38501202

[B13] Brughelli M. Cronin J. Levin G. Chaouachi A. (2008). Understanding change of direction ability in sport: a review of resistance training studies. Sports Med. Auckl. 38 (12), 1045–1063. 10.2165/00007256-200838120-00007 19026020

[B14] Chard M. D. Lachmann S. M. (1987). Racquet sports--patterns of injury presenting to a sports injury clinic. Br. J. Sports Med. 21 (4), 150–153. 10.1136/bjsm.21.4.150 3435816 PMC1478480

[B15] Cheung M. W.-L. (2014). Modeling dependent effect sizes with three-level meta-analyses: a structural equation modeling approach. Psychol. Methods 19 (2), 211–229. 10.1037/a0032968 23834422

[B16] Cheung M. W.-L. (2019). A guide to conducting a meta-analysis with non-independent effect sizes. Neuropsychol. Rev. 29 (4), 387–396. 10.1007/s11065-019-09415-6 31446547 PMC6892772

[B17] Cohen J. (1988). Statistical power analysis for the behavioral sciences. 2nd edn. New York: Routledge.

[B18] Concha-Cisternas Y. Castro-Piñero J. Leiva-Ordóñez A. M. Valdés-Badilla P. Celis-Morales C. Guzmán-Muñoz E. (2023). Effects of neuromuscular training on physical performance in older people: a systematic review. Life Basel, Switz. 13 (4), 869. 10.3390/life13040869 37109398 PMC10147025

[B19] Cumpston M. Li T. Page M. J. Chandler J. Welch V. A. Higgins J. P. (2019). Updated guidance for trusted systematic reviews: a new edition of the cochrane handbook for systematic reviews of interventions. Cochrane Database Syst. Rev. 10, ED000142. 10.1002/14651858.ED000142 31643080 PMC10284251

[B20] Daneshjoo A. Mokhtar A. H Rahnama N. Yusof A. (2013). Effects of the 11+ and Harmoknee Warm-up Programs on Physical Performance Measures in Professional Soccer Players. J. Sports Sci. 12 (3), 489–496. 10.5604/20831862.1077554 24149156 PMC3772593

[B21] de Morton N. A. (2009). The PEDro scale is a valid measure of the methodological quality of clinical trials: a demographic study. Aust. J. Physiother. 55 (2), 129–133. 10.1016/s0004-9514(09)70043-1 19463084

[B22] Deeks J. J. Higgins J. P. T. Altman D. G. Cochrane Statistical Methods Group (2019). “Analysing data and undertaking meta-analyses,” in Cochrane handbook for systematic reviews of interventions (John Wiley and Sons, Ltd), 241–284. 10.1002/9781119536604.ch10

[B23] DerSimonian R. Kacker R. (2007). Random-effects model for meta-analysis of clinical trials: an update. Contemp. Clin. Trials 28 (2), 105–114. 10.1016/j.cct.2006.04.004 16807131

[B24] DerSimonian R. Laird N. (1986). Meta-analysis in clinical trials. Control. Clin. Trials 7 (3), 177–188. 10.1016/0197-2456(86)90046-2 3802833

[B25] Dos'Santos T. Thomas C. Jones P. A. Comfort P. (2017). Mechanical determinants of faster change of direction speed performance in Male athletes. J. Strength Cond. Res. 31 (3), 696–705. 10.1519/JSC.0000000000001535 27379954

[B26] Drevon D. Fursa S. R. Malcolm A. L. (2017). Intercoder reliability and validity of WebPlotDigitizer in extracting graphed data. Behav. Modif. 41 (2), 323–339. 10.1177/0145445516673998 27760807

[B27] Dunlap W. Cortina J. Vaslow J. Burke M. J. (1996). Meta-analysis of experiments with matched groups or repeated measures designs. Psychol. Methods 1, 170–177. 10.1037/1082-989x.1.2.170

[B28] Egger M. Davey Smith G. Schneider M. Minder C. (1997). Bias in meta-analysis detected by a simple, graphical test. BMJ Clin. Research 315 (7109), 629–634. 10.1136/bmj.315.7109.629 9310563 PMC2127453

[B29] Emery C. A. Roy T.-O. Whittaker J. L. Nettel-Aguirre A. van Mechelen W. (2015). Neuromuscular training injury prevention strategies in youth sport: a systematic review and meta-analysis. Br. J. Sports Med. 49 (13), 865–870. 10.1136/bjsports-2015-094639 26084526

[B30] Fernández-Castilla B. Declercq L. Jamshidi L. Beretvas S. N. Onghena P. Van den Noortgate W. (2021). Detecting selection bias in meta-analyses with multiple outcomes: a simulation study. J. Exp. Educ. 89 (1), 125–144. 10.1080/00220973.2019.1582470 32808180

[B31] Fernandez-Fernandez J. Ulbricht A. Ferrauti A. (2014). Fitness testing of tennis players: how valuable is it? Br. J. Sports Med. 48 (Suppl. 1), i22–i31. 10.1136/bjsports-2013-093152 24668375 PMC3995228

[B32] Fernandez-Fernandez J. García-Tormo V. Santos-Rosa F. J. Teixeira A. S. Nakamura F. Y. Granacher U. (2020). The effect of a neuromuscular vs. dynamic Warm-up on physical performance in young tennis players. J. Strength and Cond. Res. 34 (10), 2776–2784. 10.1519/JSC.0000000000003703 32986392

[B33] Gibbons R. D. Hedeker D. R. Davis J. M. (1993). Estimation of effect size from a series of experiments involving paired comparisons. J. Educ. Statistics 18 (3), 271–279. 10.2307/1165136

[B34] Guney H. Yuksel I. Kaya D. Doral M. N. (2016). Correlation between quadriceps to hamstring ratio and functional outcomes in patellofemoral pain. Knee 23 (4), 610–615. 10.1016/j.knee.2016.04.004 27184883

[B35] Hachana Y. Chaabène H. Nabli M. A. Attia A. Moualhi J. Farhat N. (2013). Test-retest reliability, criterion-related validity, and minimal detectable change of the Illinois agility test in male team sport athletes. J. Strength Cond. Res. 27 (10), 2752–2759. 10.1519/JSC.0b013e3182890ac3 23439329

[B36] Hammami R. Negra Y. Nebigh A. Ramirez-Campillo R. Moran J. Chaabene H. (2023). Preseason integrative neuromuscular training improves selected measures of physical fitness in highly trained, youth, Male soccer players. J. Strength Cond. Res. 37 (6), e384–e390. 10.1519/JSC.0000000000004394 37235541

[B37] Hariton E. Locascio J. J. (2018). Randomised controlled trials - the gold standard for effectiveness research: study design: randomised controlled trials. BJOG 125 (13), 1716. 10.1111/1471-0528.15199 29916205 PMC6235704

[B38] Hedges L. V. Olkin I. (1985). “Random effects models for effect sizes,” in Statistical Methods for Meta-Analysis. Orlando, FL: Academic Press), 189–203.

[B39] Hedges L. V. Tipton E. Johnson M. C. (2010). Robust variance estimation in meta-regression with dependent effect size estimates. Res. Synthesis Methods 1 (1), 39–65. 10.1002/jrsm.5 26056092

[B40] Herman K. Barton C. Malliaras P. Morrissey D. (2012). The effectiveness of neuromuscular warm-up strategies, that require no additional equipment, for preventing lower limb injuries during sports participation: a systematic review. BMC Med. 10, 75. 10.1186/1741-7015-10-75 22812375 PMC3408383

[B41] Hopkins W. Batterham A. (2018). Improving meta-analyses in sport and exercise. Available online at: https://www.semanticscholar.org/paper/Improving-Meta-analyses-in-Sport-and-Exercise-Hopkins-Batterham/45d4703e70a1f9962ab87f6cf25fef143ccc529b (Accessed January 7, 2024).

[B42] Hung C.-L. Hung M.-H. Chang C.-Y. Wang H.-H. Ho C.-S. Lin K.-C. (2020). Influences of lateral jump smash actions in different situations on the lower extremity load of badminton players. J. Sports Sci. and Med. 19 (2), 264–270. 32390719 PMC7196745

[B43] Impellizzeri F. M. Bizzini M. Dvorak J. Pellegrini B. Schena F. Junge A. (2013). Physiological and performance responses to the FIFA 11+ (part 2): a randomised controlled trial on the training effects. J. Sports Sciences 31 (13), 1491–1502. 10.1080/02640414.2013.802926 23855764

[B44] IntHout J. Ioannidis J. P. A. Rovers M. M. Goeman J. J. (2016). Plea for routinely presenting prediction intervals in meta-analysis. BMJ Open 6 (7), e010247. 10.1136/bmjopen-2015-010247 27406637 PMC4947751

[B45] Kadlec D. Sainani K. L. Nimphius S. (2023). With great power comes great responsibility: common errors in meta-analyses and meta-regressions in strength and conditioning research. Sports Med. 53 (2), 313–325. 10.1007/s40279-022-01766-0 36208412 PMC9877053

[B46] Ko S.-H. Cha J.-R. Lee C.-C. Kim M. S. Park K. B. (2024). Musculoskeletal injuries in table tennis during competition: a systematic review. Int. J. Sports Med. 45 (4), 267–271. 10.1055/a-2175-6509 37871618 PMC10987229

[B47] Lakens D. (2013). Calculating and reporting effect sizes to facilitate cumulative science: a practical primer for t-tests and ANOVAs. Front. Psychol. 4, 863. 10.3389/fpsyg.2013.00863 24324449 PMC3840331

[B48] Lindblom H. Waldén M. Hägglund M. (2012). No effect on performance tests from a neuromuscular warm-up programme in youth female football: a randomised controlled trial. Knee Surgery, Sports Traumatology, Arthroscopy 20 (10), 2116–2123. 10.1007/s00167-011-1846-9 22203049

[B49] Liu R. Liu J. Ma X. Li Q. (2021). Effect of FIFA 11+ intervention on change of direction performance in soccer and futsal players: a systematic review and meta-analysis. Int. J. Sports Sci. and Coach. 16 (3), 862–872. 10.1177/1747954121991667

[B50] Lopes M. Rodrigues J. M. Monteiro P. Rodrigues M. Costa R. Oliveira J. (2019). Balance and proprioception responses to FIFA 11+ in amateur futsal players: short and long-term effects. J. Sports Sciences 37 (20), 2300–2308. 10.1080/02640414.2019.1628626 31200633

[B51] Lopes M. Rodrigues J. M. Monteiro P. Costa R. Oliveira J. (2020). Effects of the FIFA 11+ on ankle evertors latency time and knee muscle strength in amateur futsal players. Eur. J. Sport Sci. 20 (1), 24–34. 10.1080/17461391.2019.1609588 31092112

[B52] Lorenz D. S. Reiman M. P. Lehecka B. J. Naylor A. (2013). What performance characteristics determine elite *versus* nonelite athletes in the same sport? Sports Health 5 (6), 542–547. 10.1177/1941738113479763 24427430 PMC3806174

[B53] Lutz F. D. Cleary C. J. Moffatt H. M. Sullivan V. E. LaRoche D. P. Cook S. B. (2022). Comparison of the H:Q ratio between the dominant and nondominant legs of soccer players: a meta-analysis. Sports Health 15 (4), 486–496. 10.1177/19417381221095096 35619586 PMC10293569

[B54] Mahood Q. Van Eerd D. Irvin E. (2014). Searching for grey literature for systematic reviews: challenges and benefits. Res. Synthesis Methods 5 (3), 221–234. 10.1002/jrsm.1106 26052848

[B55] Morris S. B. (2000). Distribution of the standardized mean change effect size for meta-analysis on repeated measures. Br. J. Math. Stat. Psychol. 53 (Pt 1), 17–29. 10.1348/000711000159150 10895520

[B56] Morris S. B. (2008). Estimating effect sizes from pretest-posttest-control group designs. Organ. Res. Methods 11 (2), 364–386. 10.1177/1094428106291059

[B57] Morris S. B. DeShon R. P. (2002). Combining effect size estimates in meta-analysis with repeated measures and independent-groups designs. Psychol. Methods 7 (1), 105–125. 10.1037/1082-989x.7.1.105 11928886

[B58] Muller M. R. Lemes Í. R. Silva M. S. de C. Silva N. S. Hernández A. G. M. Pinto R. Z. (2023). The efficacy of neuromuscular training, with minimal or no equipment, on performance of youth athletes: a systematic review with meta-analysis. Phys. Ther. Sport 64, 104–116. 10.1016/j.ptsp.2023.09.010 37820456

[B59] Nakagawa S. Noble D. W. A. Senior A. M. Lagisz M. (2017). Meta-evaluation of meta-analysis: ten appraisal questions for biologists. BMC Biology 15 (1), 18. 10.1186/s12915-017-0357-7 28257642 PMC5336618

[B60] Nuhmani S. (2021). The FIFA 11+ does not alter performance in amateur female basketball players—a randomized control trial. J. Complementary Integr. Med. 18 (2), 379–383. 10.1515/jcim-2020-0081 34187120

[B61] Oliano V. J. Teixeira L. P. Lara S. Balk R. S. Fagundes S. G. (2017). Effect of FIFA 11+ in addition to conventional handball training on balance and isokinetic strength. Rev. Bras. Cineantropometria Desempenho Hum. 19 (4), 406–415. 10.5007/1980-0037.2017v19n4p406

[B62] Osawa Y. Studenski S. A. Ferrucci L. (2018). Knee extension rate of torque development and peak torque: associations with lower extremity function. J. Cachexia, Sarcopenia Muscle 9 (3), 530–539. 10.1002/jcsm.12285 29569834 PMC5989739

[B63] Page M. J. McKenzie J. E. Bossuyt P. M. Boutron I. Hoffmann T. C. Mulrow C. D. (2021). The PRISMA 2020 statement: an updated guideline for reporting systematic reviews. BMJ 372, n71. 10.1136/bmj.n71 33782057 PMC8005924

[B64] Panagoulis C. Chatzinikolaou A. Avloniti A. Leontsini D. Deli C. K. Draganidis D. (2020). In-Season integrative neuromuscular strength training improves performance of early-adolescent soccer athletes. J. Strength Cond. Res. 34 (2), 516–526. 10.1519/JSC.0000000000002938 30431535

[B65] Panchal R. Rizvi M. R. Sharma A. Ahmad F. Hasan S. Shaik A. R. (2025). Comparing the effectiveness of the FIFA 11+ warm-up and conventional warm-up in enhancing cyclist performance and mitigating injury risk. Sci. Rep. 15 (1), 9430. 10.1038/s41598-025-91005-z 40108222 PMC11923219

[B66] Paravlic A. H. Bakalár P. Puš K. Pišot S. Kalc M. Teraž K. (2024). The effectiveness of neuromuscular training warm-up program for injury prevention in adolescent male basketball players. J. Sports Sci. 42 (22), 2083–2092. 10.1080/02640414.2024.2415215 39545620

[B67] Pardos-Mainer E. Casajús J. A. Gonzalo-Skok O. (2019). Adolescent female soccer players' soccer-specific warm-up effects on performance and inter-limb asymmetries. Biol. Sport 36 (3), 199–207. 10.5114/biolsport.2019.85453 31624413 PMC6786331

[B68] Parraca J. A. Adsuar J. C. Domínguez-Muñoz F. J. Barrios-Fernandez S. Tomas-Carus P. (2022). Test-retest reliability of isokinetic strength measurements in lower limbs in elderly. Biology 11 (6), 802. 10.3390/biology11060802 35741323 PMC9219978

[B69] Pasanen K. Parkkari J. Pasanen M. Kannus P. (2009). Effect of a neuromuscular warm-up programme on muscle power, balance, speed and agility: a randomised controlled study. Br. J. Sports Med. 43 (13), 1073–1078. 10.1136/bjsm.2009.061747 19622526

[B70] Patti A. Giustino V. Cataldi S. Stoppa V. Ferrando F. Marvulli R. (2022). Effects of 5-Week of FIFA 11+ Warm-Up program on explosive strength, speed, and perception of physical exertion in elite female futsal athletes. Sports 10 (7), 100. 10.3390/sports10070100 35878111 PMC9322867

[B71] Paul D. J. Gabbett T. J. Nassis G. P. (2016). Agility in team sports: testing, training and factors affecting performance. Sports Med. 46 (3), 421–442. 10.1007/s40279-015-0428-2 26670456

[B72] Perrin D. H. Robertson R. J. Ray R. L. (1987). Bilateral lsokinetic peak torque, torque acceleration energy, power, and work relationships in athletes and nonathletes. J. Orthop. Sports Phys. Ther. 9 (5), 184–189. 10.2519/jospt.1987.9.5.184 18797005

[B73] Peters J. L. Sutton A. J. Jones D. R. Abrams K. R. Rushton L. (2008). Contour-enhanced meta-analysis funnel plots help distinguish publication bias from other causes of asymmetry. J. Clin. Epidemiol. 61 (10), 991–996. 10.1016/j.jclinepi.2007.11.010 18538991

[B74] Pomares-Noguera C. Ayala F. Robles-Palazón F. J. Alomoto-Burneo J. F. López-Valenciano A. Elvira J. L. L. (2018). Training effects of the FIFA 11+ kids on physical performance in youth football players: a randomized control trial. Front. Pediatr. 6, 40. 10.3389/fped.2018.00040 29556489 PMC5844920

[B75] Pustejovsky J. E. Tipton E. (2022). Meta-analysis with robust variance estimation: expanding the range of working models. Prev. Sci. 23 (3), 425–438. 10.1007/s11121-021-01246-3 33961175

[B76] Quintana D. S. (2023). A guide for calculating study-level statistical power for meta-analyses. Adv. Methods Pract. Psychol. Sci. 6 (1), 25152459221147260. 10.1177/25152459221147260

[B77] Reis I. Rebelo A. Krustrup P. Brito J. (2013). Performance enhancement effects of Fédération Internationale de Football Association's ‘The 11+' injury prevention training program in youth futsal players. Clin. J. Sport Med. Official J. Can. Acad. Sport Med. 23 (4), 318–320. 10.1097/JSM.0b013e318285630e 23528840

[B78] Ruas C. V. Pinto R. S. Haff G. G. Lima C. D. Pinto M. D. Brown L. E. (2019). Alternative methods of determining Hamstrings-to-Quadriceps ratios: a comprehensive review. Sports Med. - Open 5 (1), 11. 10.1186/s40798-019-0185-0 30911856 PMC6434009

[B79] Ruppar T. (2020). Meta-analysis: how to quantify and explain heterogeneity? Eur. J. Cardiovasc. Nurs. 19 (7), 646–652. 10.1177/1474515120944014 32757621

[B80] Sannicandro I. Cofano G. D’Onofrio R. (2023). The effects of 8 weeks of integrative neuromuscular pitch training on strength values and sprint performance in young élite soccer players. J. Phys. Educ. Sport 23 (4), 909–917. 10.7752/jpes.2023.04114

[B81] Schünemann H. J. Higgins J. P. T. Vist G. E. Glasziou P. Akl E. A. Skoetz N. (2019). “Completing ‘Summary of findings’ tables and grading the certainty of the evidence,” in Cochrane handbook for systematic reviews of interventions (John Wiley and Sons, Ltd), 375–402. 10.1002/9781119536604.ch14

[B82] Shadish W. R. Clark M. H. Steiner P. M. (2008). Can nonrandomized experiments yield accurate answers? A randomized experiment comparing random and nonrandom assignments. J. Am. Stat. Assoc. 103 (484), 1334–1344. 10.1198/016214508000000733

[B83] Sheppard J. M. Young W. B. (2006). Agility literature review: classifications, training and testing. J. Sports Sci. 24 (9), 919–932. 10.1080/02640410500457109 16882626

[B84] Singh U. Leicht A. S. Connor J. D. Brice S. M. Alves A. Doma K. (2025). Biomechanical determinants of change of direction performance: a systematic review. Sports Med. Auckl. N.Z. 55 (9), 2207–2224. 10.1007/s40279-025-02278-3 40668491 PMC12476310

[B85] Spineli L. M. Pandis N. (2020). Prediction interval in random-effects meta-analysis. Am. J. Orthod. Dentofac. Orthop. 157 (4), 586–588. 10.1016/j.ajodo.2019.12.011 32241366

[B86] Spiteri T. Nimphius S. Hart N. H. Specos C. Sheppard J. M. Newton R. U. (2014). Contribution of strength characteristics to change of direction and agility performance in female basketball athletes. J. Strength Cond. Res. 28 (9), 2415–2423. 10.1519/JSC.0000000000000547 24875426

[B87] Steib S. Rahlf A. L. Pfeifer K. Zech A. (2017). Dose-response relationship of neuromuscular training for injury prevention in youth athletes: a meta-analysis. Front. Physiology 8, 920. 10.3389/fphys.2017.00920 29184511 PMC5694483

[B88] Sterne J. A. C. Sutton A. J. Ioannidis J. P. A. Terrin N. Jones D. R. Lau J. (2011). Recommendations for examining and interpreting funnel plot asymmetry in meta-analyses of randomised controlled trials. BMJ Clin. Research 343, d4002. 10.1136/bmj.d4002 21784880

[B89] Sterne J. A. Hernán M. A. Reeves B. C. Savović J. Berkman N. D. Viswanathan M. (2016). ROBINS-I: a tool for assessing risk of bias in non-randomised studies of interventions. BMJ 355, i4919. 10.1136/bmj.i4919 27733354 PMC5062054

[B90] Sterne J. A. C. Savović J. Page M. J. Elbers R. G. Blencowe N. S. Boutron I. (2019). RoB 2: a revised tool for assessing risk of bias in randomised trials. BMJ 366, l4898. 10.1136/bmj.l4898 31462531

[B91] Thomas C. Dos'Santos T. Comfort P. Jones P. A. (2018). Relationships between unilateral muscle strength qualities and change of direction in adolescent team-sport athletes. Sports 6 (3), 83. 10.3390/sports6030083 30127300 PMC6162690

[B92] Tipton E. Pustejovsky J. E. (2015). Small-sample adjustments for tests of moderators and model fit in robust variance estimation in meta-regression. J. Educ. Behav. Stat. 40 (6), 604–634. 10.3102/1076998615606099

[B93] Tomaras E. K. MacIntosh B. R. (2011). Less is more: standard warm-up causes fatigue and less warm-up permits greater cycling power output. J. Appl. Physiology 111 (1), 228–235. 10.1152/japplphysiol.00253.2011 21551012

[B94] Trajković N. Gušić M. Molnar S. Mačak D. Madić D. M. Bogataj Š. (2020). Short-term FIFA 11+ improves agility and jump performance in young soccer players. Int. J. Environ. Res. Public Health 17 (6), 2017. 10.3390/ijerph17062017 32197538 PMC7142544

[B95] Van den Noortgate W. López-López J. A. Marín-Martínez F. Sánchez-Meca J. (2013). Three-level meta-analysis of dependent effect sizes. Behav. Res. Methods 45 (2), 576–594. 10.3758/s13428-012-0261-6 23055166

[B96] van Dyk N. Bahr R. Whiteley R. Tol J. L. Kumar B. D. Hamilton B. (2016). Hamstring and quadriceps isokinetic strength deficits are weak risk factors for hamstring strain injuries: a 4-Year cohort study. Am. J. Sports Med. 44 (7), 1789–1795. 10.1177/0363546516632526 27002102

[B97] Viechtbauer W. (2010). Conducting meta-analyses in R with the metafor package. J. Stat. Softw. 36, 1–48. 10.18637/jss.v036.i03

[B98] Wang B. B. Wu B. Yang Y. Cai M. Li S. Peng H. (2024). Neuromuscular and balance adaptations following acute stretching exercise: a randomized control trial. Front. Physiology 15, 1486901. 10.3389/fphys.2024.1486901 39691093 PMC11649666

[B99] Wang P. Liu Y. Chen C. (2024). Effects of neuromuscular training on dynamic balance ability in athletes: a systematic review and meta-analysis. Heliyon 10 (16), e35823. 10.1016/j.heliyon.2024.e35823 39220942 PMC11365420

[B100] Wilson C. (2020). Mechanisms underpinning an improvement in dynamic muscle force production following a high-intensity warm-up. Theses: Doctorates and Masters.

[B101] Wilson C. J. Nunes J. P. Blazevich A. J. (2025). The effect of muscle warm-up on voluntary and evoked force-time parameters: a systematic review and meta-analysis with meta-regression. J. Sport Health Sci. 14, 101024. 10.1016/j.jshs.2025.101024 39864808 PMC12357318

[B102] Young W. B. James R. Montgomery I. (2002). Is muscle power related to running speed with changes of direction? J. Sports Med. Phys. Fit. 42 (3), 282–288. 12094116

[B103] Zouhal H. Abderrahman A. B. Dupont G. Truptin P. Le Bris R. Le Postec E. (2019). Effects of neuromuscular training on agility performance in elite soccer players. Front. Physiology 10, 947. 10.3389/fphys.2019.00947 31396107 PMC6664050

